# One-pot efficient synthesis of *N*^α^-urethane-protected β- and γ-amino acids

**DOI:** 10.1007/s00726-012-1443-3

**Published:** 2012-12-19

**Authors:** Marta Cal, Mariusz Jaremko, Łukasz Jaremko, Piotr Stefanowicz

**Affiliations:** 1 Faculty of Chemistry, University of Wrocław, ul. F. Joliot-Curie 14, Wroclaw, Poland; 2 Institute of Biochemistry and Biophysics, Polish Academy of Sciences, ul. Pawińskiego 5a, 02-106 Warsaw, Poland; 3 Faculty of Chemistry, Warsaw University, ul. Pasteura 1, 02-093 Warsaw, Poland

**Keywords:** *N*-Hydroxyimides, Lossen rearrangement, β- and γ-Amino acids, *N*^α^-Urethane-protection, CBz, GABA, β-Alanine, Anthranilic acid

## Abstract

1-[(4-Methylphenyl)oxy]pyrrolidine-2,5-dione and 1-[(4-methylphenyl)oxy]piperidine-2,6-dione react in a Lossen-type reaction with primary alcohols in the presence of triethylamine to furnish corresponding *N* ^α^-urethane-protected β-alanine and γ-aminopropionic acid (GABA), respectively, with excellent yields and purities, in an essentially “one-pot” procedure.

## Introduction

**Fig. 1:**
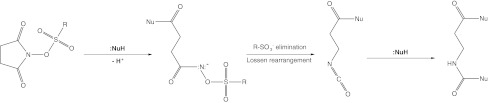
A proposed reaction mechanism between the sulfonic esters of *N*-hydroxyimides and nucleophile (:NuH, e.g. alcohols, amines) (Groutas et al. 1986)

Sulfonic esters of *N*-hydroxyimides are well known for their inhibitory properties against serine proteases, mainly human leukocyte elastase (HLE) (Abell and Oldham 1999; Groutas et al. 1986, 1992, 1993, 1994; Neumann and Guetschow 1994; Tirouvanziam 2006). It has been elucidated that the mechanism of the proteases inhibition is based on the Lossen-like rearrangement (Groutas et al. 1986), which is induced by the nucleophilic amino acid residues present in the enzyme’s active center, like the hydroxyl side group of serine. Moreover, many investigations have shown that sulfonic esters of *N*-hydroxyimides are characterized also by the specific chemical reactivity toward nucleophilic compounds, like amines (Bauer and Exner 1974; Youssef and Abbady 1997; Abbady et al. 2000), hydrazine (Youssef and Abbady 1997; Abbady et al. 2000) and alcohols (Youssef and Abbady 1997; Chandrasekhar and Sridhar 2000; Sheikh et al. 2010). These properties may be at least partially explained as a result of flattened pyramidal geometry of the succinic ring’s nitrogen atom (Stefanowicz et al. 2006). This structural feature is common for every described sulfonic ester of *N*-hydroxyimide (Stefanowicz et al. 2005, 2006, 2007). The Lossen-like reaction between the nucleophiles and sulfonic esters of *N*-hydroxyimides (Fig. 1) opens a new route for the efficient one-pot synthesis of *N* ^α^-urethane-protected compounds. Since the urethane groups are often used as protective groups, the rearrangement products may be applied as building blocks in the synthesis of peptidomimetics.

We present here such a method of synthesis of *N* ^α^-urethane-protected β-alanine and GABA and also anthranilic acid derivatives, based on the nucleophiles which induce the Lossen-like rearrangement.

β-Alanine is an essential non-proteinaceous amino acid, which can be considered as an important element of some peptidic (English et al. 2006; Koyack and Cheng 2006; Kritzer et al. 2005; Patch and Barron 2002; Porter et al. 2005; Potocky et al. 2005; Qiu et al. 2006) and peptidomimetic chains (Ranganathan et al. 1997). β-Amino acids-containing peptides exhibit well-defined secondary structures in solution, such as helices, turns, and sheets. Such peptides can also mimic a γ-turn conformation, which occurs in the case of the *cis*-proline residue (Ahmed et al. 2007; Cheng et al. 2001; Daura et al. 2001; Langenhan et al. 2003). The GABA regulates the growth of embryonic and neural stem cells. GABA activates the GABA_A_ receptor, causing cell cycle arrest in the S-phase, limiting their growth (Wang et al. 2008).

## Experimental section

Investigated compounds were synthesized according to the procedures described below. Melting points (uncorrected) were measured with a Boethius PHMK (VEB Analytik, Dresden, Deutschland) apparatus. The ^1^H and ^13^C NMR spectra were recorded on a 500 MHz Bruker spectrometer using TMS as the internal standard. Mass spectra were obtained on a micrOTOF-Q—Bruker Daltonic instrument equipped with an electrospray ion source. MeOH containing 10^−4^ M NaCl or 10^−2^ M NH_4_HCO_3_ was used as a solvent for ESI–MS measurements.

All solvents and reagents were used as supplied. Methanol, ethyl acetate, benzyl alcohol, hydrochloric acid and triethylamine (TEA) were obtained from Sigma-Aldrich. Methanol, used for ESI–MS measurements, was LC–MS quality and obtained from Merck Millipore. Solvents used for NMR experiments (CDCl_3_ and DMSO-d_6_) were obtained from Cambridge Isotope Laboratories.

### General procedure for synthesis of sulfonic esters of *N*-hydroxyimides (Stefanowicz et al. 2005, 2006, 2007; Sheikh et al. 2010)

*N*-Hydroxyimide (93 mmol) and chloride of sulfonic acid (100 mmol) were dissolved in tetrahydrofuran (100 ml) and then triethylamine (14.70 ml) was added dropwise for 20 min. After 40 min, the solvent was removed in vacuo and 100 ml of 5 % hydrochloric acid was added. The product was filtered off, washed twice with water and crystallized from ethyl acetate to give corresponding sulfonic ester of *N*-hydroxyimide.

1-{[(4-Methylphenyl)sulfonyl]oxy}pyrrolidine-2,5-dione (Stefanowicz et al. 2006) (TosOSu) **1a**. mp = 143–144 °C, lit (Stefanowicz et al. 2006) 135–136 °C (Sheikh et al. 2010). ESI–MS: Found: 292.02; calculated for (C_11_H_11_NO_5_S + Na)^+^ 292.03, ^1^H NMR (CDCl_3_, 500 MHz) d 2.44 (s, 3H), 2.76 (s, 4H), 7.35 (d, 2H, *J* = 8.4 Hz), 7.87 (d, 2H, *J* = 8.4 Hz). 92 % yield.

1-{[(4-Methylphenyl)sulfonyl]oxy}piperidine-2,6-dione (TosOGlt) **1b**. mp = 148–148,5 °C, lit (Sheikh et al. 2010) 145.5–146 °C. ESI–MS: Found: 306.04; calculated for (C_12_H_13_NO_5_S + Na)^+^ 306.04. ^1^H NMR (DMSO-d_6_, 500 MHz) δ 1.87 (q, 2H, *J* = 6.37 Hz), 2.44 (s, 3H), 2.73 (t, 4H, *J* = 6.40 Hz), 7.48 (d, 2H, *J* = 8.30 Hz), 7.85 (d, 2H, *J* = 8.30 Hz). ^13^C NMR (DMSO-d_6_, 125 MHz) δ 15.8 (CH_3_), 32.7 (C=OCH_2_), 39.7 (CH_2_), 168.0 (CH_2_C=O). Yield 70 %.

1-[(Methylsulfonyl)oxy]pyrrolidine-2,5-dione (MesOSu) **1c**. mp = 157–158 °C, lit (Stefanowicz et al. 2007) 157–158 °C. ESI–MS: Found: 216.00; calculated for (C_5_H_7_NO_5_S + Na)^+^ 215.99. ^1^H NMR (DMSO-d_6_, 500 MHz) δ 2.77 (t, 4H, *J* = 7.06 Hz), 3.59 (s, 3H). ^13^C NMR (DMSO-d_6_, 125 MHz) δ 25.5 (CH_3_), 39.7 (CH_2_CH_2_), 170.2 (C=O). Yield 64 %.

1-[(Methylsulfonyl)oxy]piperidine-2,6-dione (MesOGlt) **1d**. mp = 138–139 °C, ESI–MS: Found: 230.00; calculated for (C_6_H_9_NO_5_S + Na)^+^ 230.01. ^1^H NMR (DMSO-d_6_, 500 MHz) δ 1.90 (q, 2H, *J* = 6.36), 2.82 (t, 4H, *J* = 6.42), 3.47 (s, 3H). ^13^C NMR (DMSO-d_6_, 125 MHz) δ 15.8 (CH_3_), 32.7 (C=OCH_2_), 39.7 (CH_2_), 168.0 (CH_2_C=O). Yield 65 %.

2-{[(4-Methylphenyl)sulfonyl]oxy}-1H-isoindole-1,3(2H)-dione (TosOPh) **1e**. mp = 155–157 °C, lit (Stefanowicz et al. 2006) 154–157 °C, lit (Sheikh et al. 2010) 161.0–161.5 °C. ESI–MS: Found: 340.02; calculated for (C1_5_H_11_NO_5_S + Na)^+^ 340.03. ^1^H NMR (CDCl_3_, 500 MHz) d 2.48 (s, 3H), 7.39 (d, 2H, *J* = 8.2 Hz), 7.78 (m, 2H), 7. 85 (m, 2H) 7.93 (d, 2H, *J* = 8.3 Hz)., 91 % yield.

### Synthesis of *N*-urethane-protected amino acids

#### Benzyl alcohol as a reaction substrate and as a solvent (compound 2a-c)

Sulfonic esters of *N*-hydroxyimides (**1a-d**, 1.0 g) were dissolved in 4.5 ml of benzyl alcohol. Later 2.2 ml of triethylamine (TEA) was added dropwise to the solution. The reaction mixtures were stirred at 65 °C for 1 h. The reaction product was diluted with 60 ml of methanol. Next 5 ml of 1 M methanolic NaOH solution was added and the mixtures were incubated for 1 h. The solvent was removed in vacuo and residue was dissolved in 50 ml of water. The solution was acidified to pH 1 with 5 N HCl. The product, which partially had crystallized, was extracted with ethyl acetate and solvent was evaporated in vacuo.

#### Trifluoroethanol as a substrate and as a solvent (compound 3a,b)

TosOSu or TosOGlt (**1a**, **b** 1.0 g) was dissolved in 4.5 ml of benzyl alcohol. TEA (triethylamine; 2.2 ml) was added dropwise to the solution. The reaction mixture was stirred at 65 °C for 1 h. The excess of alcohol was removed in vacuum. The reaction product was extracted with ethyl acetate, washed with 5 % hydrochloric acid and water. The organic layer was evaporated in vacuo to obtain oily residue.

#### 4-Chlorobenzyl alcohol and 4-methoxybenzyl alcohol as a substrate and as a solvent (compound 4a,b and 5a,b, respectively)

TosOSu or TosOGlt (**1a,b** 1,0 g) was added to 4.5 g/4.5 g of 4-chlorobenzyl/4-methoxybenzyl alcohol. Later 2.2 ml of TEA was added dropwise. The reaction mixture was stirred at 65 °C for 1 h. The reaction gives a product which was then diluted with 60 ml of methanol and hydrolyzed with the addition of 5 ml of 1 M methanolic NaOH solution. Then the reaction mixture was acidified to pH 1 with 5 N HCl. The product was extracted with ethyl acetate which was subsequently removed in vacuo.

#### Benzene as a solvent

TosOSu or TosOGlt (**1a,b**; 1.0 g) was dissolved in 10 ml of benzene and 2.5 ml of benzyl alcohol was added. Later 1.5 ml of TEA was added dropwise. The reaction mixture was stirred at 65 °C for 1 h. Then the mixture was diluted with 30 ml of methanol and after addition of 2.5 ml of 1 M methanolic NaOH solution was incubated for 1 h. The solvent was removed in vacuo and the residue was dissolved in 25 ml of water. After being acidified to pH 1 with 5 N HCl, the partially crystallized product was extracted with ethyl acetate. The solvent was evaporated in vacuo. The residue was dissolved in 25 ml of water and acidified to pH 1 with 5 N HCl. The product, which partially had crystallized, was extracted with ethyl acetate and solvent was evaporated in vacuo.

### Analytical data for obtained products

4-{[(Benzyloxy)carbonyl]amino}propanoic acid (N-[(benzyloxy)carbonyl]-beta-alanine) **(2a)**. mp = 99–100 °C. ESI–MS: Found: 222.07; calculated for (C_11_H_13_NO_4_–H)^−^ 222.08. ^1^H NMR (500 MHz, CDCl_3_): δ = 2.58 ppm (t, *J* = 6.85 Hz, 2H); 3.44 ppm (q, *J* = 5.10 Hz, 2H); 5.00 ppm (s, 2H); 5.30 ppm (broad signal, 0.78H); 6.31 ppm (broad signal, 0.22H); 7.29–7.34 ppm (m, 5H); 9.49 ppm (broad signal. 1H). ^1^H NMR (500 MHz, DMSO-d6): δ = 2.39 ppm (t, *J* = 6.72 Hz, 2H); 3.20 ppm (m, 2H); 5.08 ppm (s, 2H); 7.27 ppm (t, *J* = 5.30 Hz, 1H); 7.29–7.34 ppm (m, 5H).

4-{[(Benzyloxy)carbonyl]amino}butanoic acid (**2b**). mp = 61–62 °C. ESI–MS: Found: 236.09; calculated for (C_12_H_15_NO_4_–H)^−^ 236.19. ^1^H NMR (500 MHz, CDCl_3_): δ = 1.83 ppm (m, *J* = 6.92 Hz, 2H); 2.39 ppm (t, *J* = 7.00 Hz, 2H); 3.25 ppm (q, *J* = 6.42 Hz, 2H); 4.88 ppm (s, 1H); 5.08 ppm (s, 2H); 7.29–7.34 ppm (m, 5H); 9.49 ppm. ^1^H NMR (500 MHz, DMSO-d_6_): δ = 1.61 ppm (m, 2H); 2.20 ppm (t, *J* = 9.58 Hz, 2H); 3.00 ppm (q, *J* = 6.02 Hz, *J* = 6.13 Hz, 2H); 4.99 ppm (s, 2H); 7.31 ppm (d, *J* = 7.42 Hz, 2H); 7.32-7.35 ppm (m, 5H); 9.49 ppm.

2-{[(Benzyloxy)carbonyl]amino}benzoic acid (**2c**). mp = 110–112 °C. ESI–MS: Found: 270.07; calculated for (C_15_H_13_NO_4_–H)^−^ 270.08. ^1^H NMR (500 MHz, DMSO-d_6_): δ = 5.17 ppm (broad signal, 2H).; δ = 7.10 ppm (m, 1H); 7.33–7.43 (m, 5H) ppm; 7.59 ppm (t, *J* = 8.53 Hz, 1H); 7.96 ppm (d, *J* = 7.96 Hz, 1H); 8.26 ppm (t, *J* = 8.26 Hz, 1H).

2,2,2-Trifluoroethyl 3-{[(2,2,2-trifluoroethoxy)carbonyl]amino}propanoate (**3a**). mp = 33 °C. ESI–MS: Found: 320.03; calculated for (C_8_H_9_F_6_NO_4_ + Na)^+^ 320.02. ^1^H NMR (500 MHz, CDCl_3_): δ = 2.64 ppm (t, *J* = 6.08 Hz, 2H); 3.46 ppm (q, *J* = 6.11 Hz, 2H); 4.39 ppm (q, 3 J = 8.52 Hz, 2H); 4.44 ppm (q, *J* = 8.42 Hz, 2H); 5.68 ppm (broad signal, 1H). ^1^H NMR (500 MHz, DMSO-d_6_): δ = 2.61 ppm (t, *J* = 6.73 Hz, 2H); 3.26 ppm (q, *J* = 6.96 Hz, 1H); 4.63 ppm (q, 3 J = 9.04 Hz, 2H); 7.74 ppm (broad signal, 1H).

2,2,2-Trifluoroethyl 4-{[(2,2,2-trifluoroethoxy)carbonyl]amino}butanoate (**3b**). mp = 30 °C. ESI–MS: Found: 334.05; calculated for (C_9_H_11_F_6_NO_4_ + Na)^+^ 334.05. ^1^H NMR (500 MHz, CDCl_3_): δ = 1.84 ppm (m, *J* = 7.06 Hz, 2H); 2.43 ppm (t, *J* = 7.30 Hz, 2H); 3.23 ppm (q, 3 J = 6.57 Hz, 2H); 4.38–4.44 ppm (m, 3 Hz, 4H); 5.40 ppm (broad signal, 1H). ^1^H NMR (500 MHz, DMSO-d_6_): δ = 1.73 ppm (m, *J* = 7.18 Hz, 2H); 2.44 ppm (t, *J* = 7.46 Hz, 2H); 3. 06 ppm (q, *J* = 6.01 Hz, 2H); 4.62 ppm (q, *J* = 9.12 Hz); 7.65 ppm (broad signal, 1H).

3-({[(4-Methoxybenzyl)oxy]carbonyl}amino)propanoic acid (**5a**). mp = 79–81 °C. ESI–MS: Found: 252.09; calculated for (C_12_H_15_NO_5_–H)^−^ 252.09. ^1^H NMR (500 MHz, CDCl_3_): δ = 2.56 ppm (t, *J* = 2 Hz, 2H); 3.42 ppm (multiplet, 2H); 3.78 ppm (s, 3H); 5.00 ppm (s, 0.56H); 5.06 ppm (s, 1.44H); 5.28 ppm (s, 0.72H); 6.29 ppm (s, 0.28H); 6.85 ppm (d, *J* = 7.89 Hz, 2H); 7.26 ppm (d, *J* = 7.90 Hz, 2H); 8.70–9.65 ppm (broad signal, 1H). ^1^H NMR (500 MHz, DMSO-d_6_): δ = 2.34 ppm (t, *J* = 6.94 Hz, 2H); 3.15 ppm (quartet, *J* = 6.60, *J* = 6.28, 2H); 3.70 ppm (s, 3H); 4.88 ppm (s, 2H); 6.87 ppm (d, *J* = 8.41 Hz, 2H); 7.16 (t, *J* = 6.18 Hz, 1H); 7.24 ppm (d, *J* = 8.41 Hz, 2H).

4-({[(4-Methoxybenzyl)oxy]carbonyl}amino)butanoic acid (**5b**). mp = (liquid at RT). ESI–MS: Found: 266.10; calculated for (C_13_H_17_NO_5_–H)^−^ 266.09. ^1^H NMR (500 MHz, CDCl_3_): δ = 1.94 ppm (m, *J* = 7.83 Hz, *J* = 7.74 Hz, 2H); 2.39 ppm (t, *J* = 6.33 Hz, 2H); 2.80 ppm (q, *J* = 7.48 Hz, 2H); 3.69 ppm (s, 3H); 4.50 ppm (s, 2H); 6.89 ppm (d, *J* = 7.20 Hz, 2H); 7.36 ppm (d, *J* = 7.70 Hz, 2H); 5.86 ppm (broad signal, 1H). ^1^H NMR (500 MHz, DMSO-d_6_): δ = 1.72 ppm (m, *J* = 7.33 Hz, *J* = 7.54 Hz, 2H); 2.29 ppm (t, *J* = 7.30 Hz, 2H); 2.77 ppm (q, *J* = 7.28 Hz, 2H); 3.63 ppm (s, 3H); 4.57 ppm (s, 2H); 7.09 ppm (d, *J* = 7.70 Hz, 2H); 7.45 ppm (d, *J* = 7.75 Hz, 2H); 7.66 ppm (broad signal, 1H).

3-({[(4-Chlorobenzyl)oxy]carbonyl}amino)propanoic acid (**4a**). mp = 119–120° C. ESI–MS: Found: 256.04; calculated for (C_12_H_14_ClNO_4_–H)^−^ 256.04. ^1^H NMR (500 MHz, CDCl_3_): δ = 2.61 ppm (t, *J* = 3 Hz, 2H); 3.48 ppm (m, 2H); 5.07 ppm (s, 2H); 5.28-542 ppm (broad signal, 0.89H); 6.18–6.30 ppm (broad signal, 0.11H); 7.28 ppm (d, 3 J = 8.40 Hz, 2H); 7.33 ppm (d, *J* = 8.40 Hz, 2H). ^1^H NMR (500 MHz, DMSO-d_6_): δ = 2.37 ppm (t, *J* = 6.76 Hz, 2H); 3.19 ppm (q, *J* = 6.28, 3 J = 6.18, 2H); 4.98 ppm (s, 2H); 7.31 ppm (t, *J* = 6.18 Hz, 1H); 7.36 ppm (d, *J* = 7.39 Hz, 2H); 7.41 ppm (d, *J* = 7.41 Hz, 2H).

4-({[(4-Chlorobenzyl)oxy]carbonyl}amino)butanoic acid (**4b**). mp = 100° C. ESI–MS: Found: 270.05; calculated for (C_12_H_14_ClNO_4_–H)^−^ 270.06. ^1^H NMR (500 MHz, CDCl_3_): δ = 1.86 ppm (m, *J* = 6.96 Hz, 2H); 2.41 ppm (t, *J* = 7.10 Hz, 2H); 3.27 ppm (t, *J* = 6.75 Hz, 2H); 5.08 ppm (s, 2H); 5.90–5.08 ppm (broad signal, 0.10H); 7.05–7.25 ppm (broad signal, 0.90H); 7.29 ppm (d, *J* = 8.40 Hz, 2H); 7.33 ppm (d, *J* = 8.40 Hz, 2H). ^1^H NMR (500 MHz, DMSO-d_6_): δ = 1.52 ppm (m, *J* = 6.79 Hz, *J* = 6.77 Hz, 2H); 2.11 ppm (t, *J* = 7.02 Hz, 2H); 2.91 ppm (q, *J* = 5.73 Hz, 2H); 4.90 ppm (s, 2H); 7.21 ppm (t, *J* = 5.90 Hz, 1H); 7.27 ppm (d, *J* = 7.80 Hz, 2H); 7.32 ppm (d, *J* = 7.81 Hz, 2H).

## Results and discussion

β and γ dicarboxylic acids may be relatively easily converted to cyclic *N*-hydroxyimides. These cyclization products are often used as the additives in peptide synthesis and for activation of carboxylic group in the modification of proteins. *N*-hydroxyimides also react with aromatic and aliphatic sulfochlorides forming crystalline derivatives with high yield. In this paper, we tested a new way of synthesis of N-protected non-proteinaceous amino acids based on Lossen rearrangement. Reaction of sulfonic ester of *N*-hydroxyimide and selected alcohol is carried out in the presence of triethylamine at 65 °C. As the solvents we tested pyridine, benzene and the alcohol itself.

**Fig. 2:**
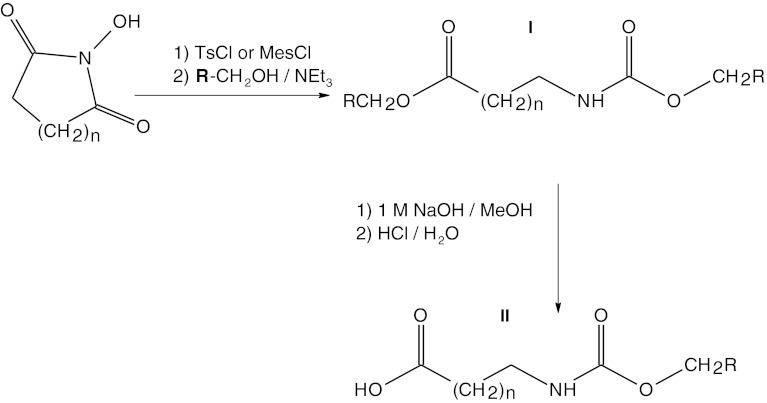
Scheme of the reaction of obtaining *N* ^α^-urethane-protected β-alanine and γ-aminopropionic acid (GABA) derivatives

The aryl- and alkylsulfonates of hydroxyimides form the product **I**, which is next hydrolyzed with 1 M methanolic NaOH solution forming *N*-urethane-protected amino acid. Then the reaction mixture is acidified to pH 1 with 5 M HCl and the product **II** crystallizes spontaneously or is extracted with ethyl acetate which is subsequently removed in vacuo (Fig. 2). The yield of β-alanine derivatives synthesized by this procedure is in the range of 38–82 %. The lowest yield is obtained when benzene is used as a solvent (38 %), the highest—when 4-methoxybenzyl alcohol is used (82 %). However, all the results are much higher than those obtained for other reported methods of synthesis of β-alanine-containing compounds (Ranganathan et al. 1997).

The same procedure was also tested on secondary and tertiary alcohols; however, the attempt on the isolation of reaction product was unsuccessful. The obtained results show clearly that the rearrangement occurs only with primary alcohols, like benzyl alcohol and its derivatives. Performing the reaction under reflux with the addition of pyridine as a base does not give any rearrangement products either.

Reactions were also performed in a microwave oven using conditions analogical to those shown above; however, the yields were not higher than when conventional heating was used (data not presented).

Application of triethylamine as a base in benzene solvent results in the yields which are in the range of 31–38 % (Table 1). The highest reaction yield was obtained in alcohol, which plays a double role as a solvent and as a reaction substrate, in presence of triethylamine (Table 1).

**Table 1:**
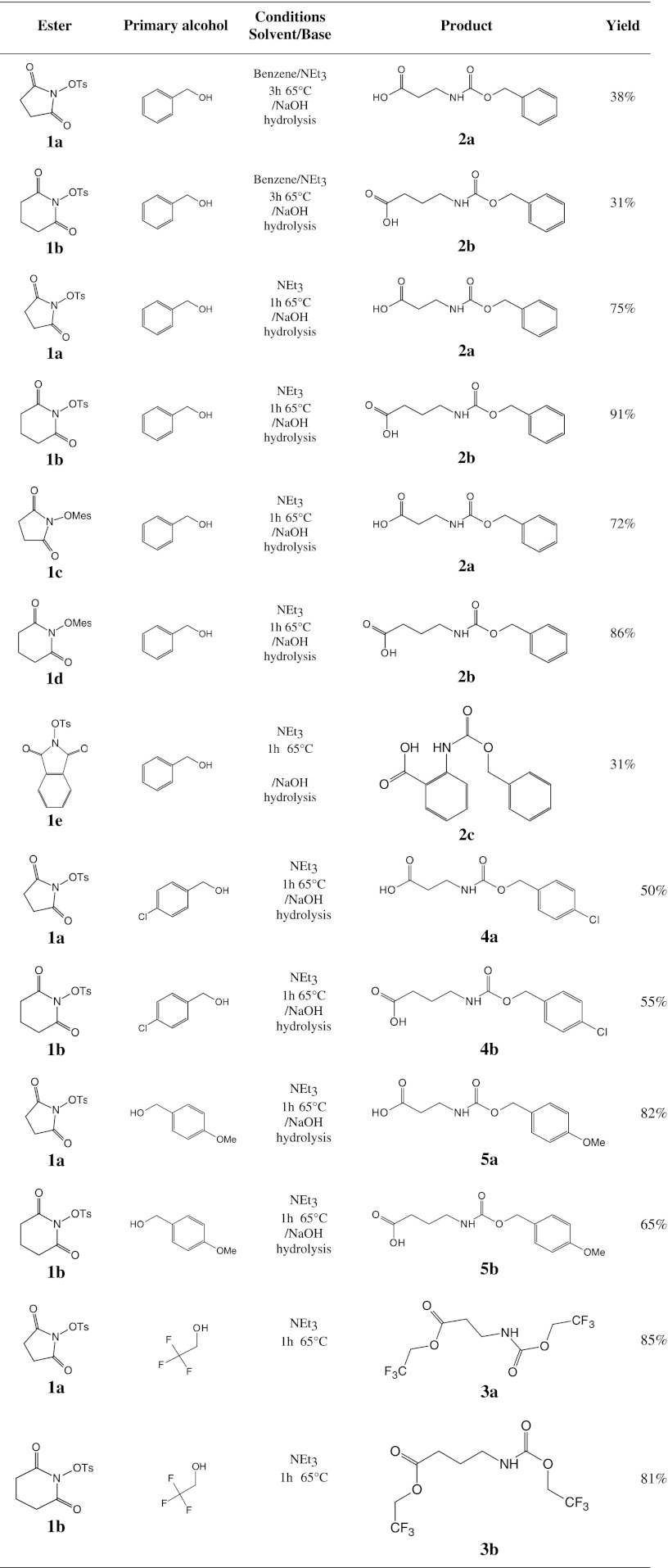
The yields of synthesis of *N*-α-urethane-protected β- and γ-amino acids

**Fig. 3:**
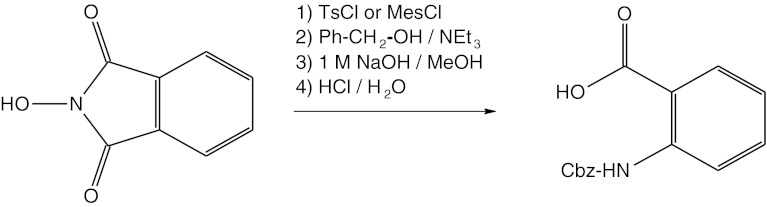
Scheme of the reaction between *N*-hydroxyphthalimide and benzyl alcohol

The reaction described in this paper can also be used for synthesis of anthranilic acid *N*-urethane-protected derivatives (Fig. 3). However, the yield of this reaction does not exceed 31 %.

## Conclusion

The simple one-pot procedure proposed in this study provides a new less time-consuming and relatively cheap way of obtaining *N* ^α^-urethane-protected β-alanine and γ-aminopropionic acid (GABA) derivatives from stable and easily available substances, like primary alcohols and sulfonic esters of *N*-oxyimides. Reaction is limited to primary alcohols. The crude products obtained in this route are ready for further synthesis without additional purification.
